# Supplementary material for: Prognostic Value of Fusobacterium nucleatum after Abdominoperineal Resection for Anal Squamous Cell Carcinoma
Source: Cancers (Basel). 2022 Mar 22;14(7):1606. doi: 10.3390/cancers14071606 (PMC8997094; doi:10.3390/cancers14071606)
Supplement: Supplementary file 1 [file cancers-14-01606-s001.zip › cancers-1617437-supplementary.pdf]

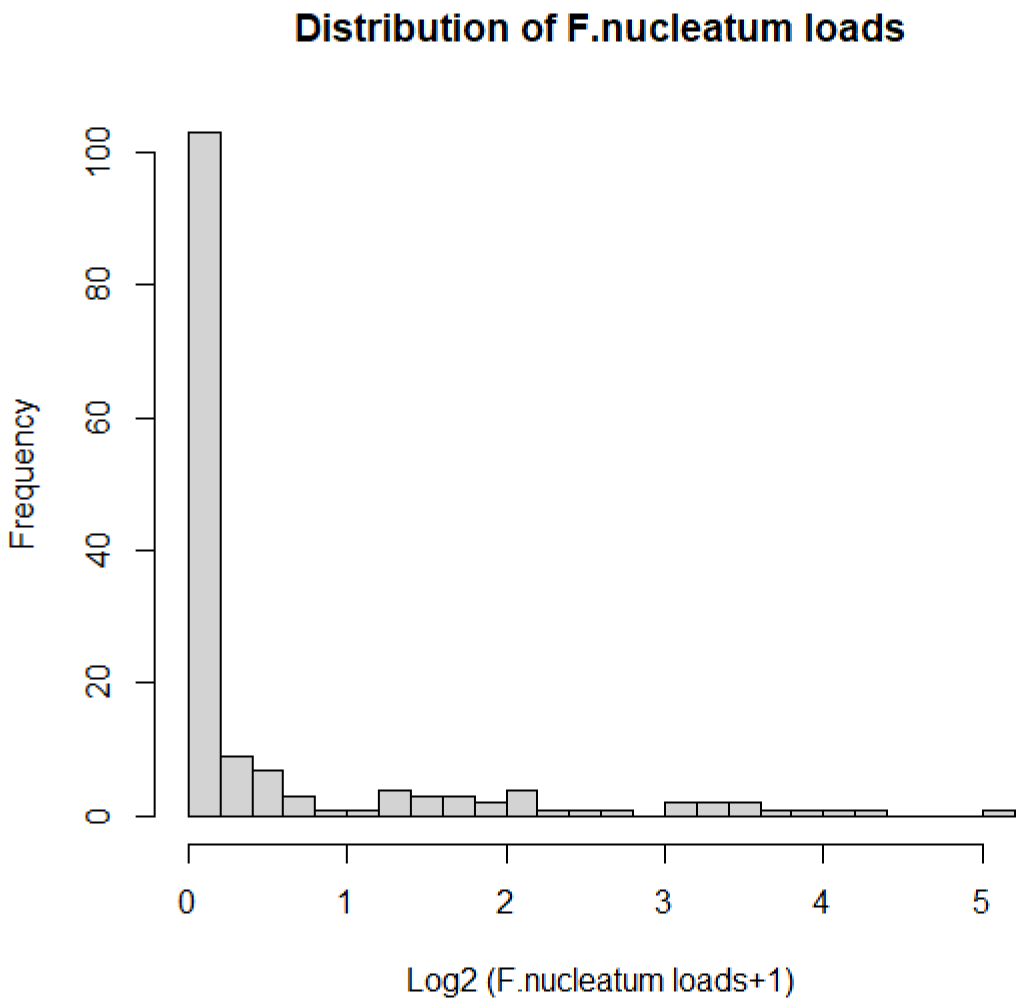

Figure S1. Distribution of normalized and logged Fusobacterium loads in the patient population.

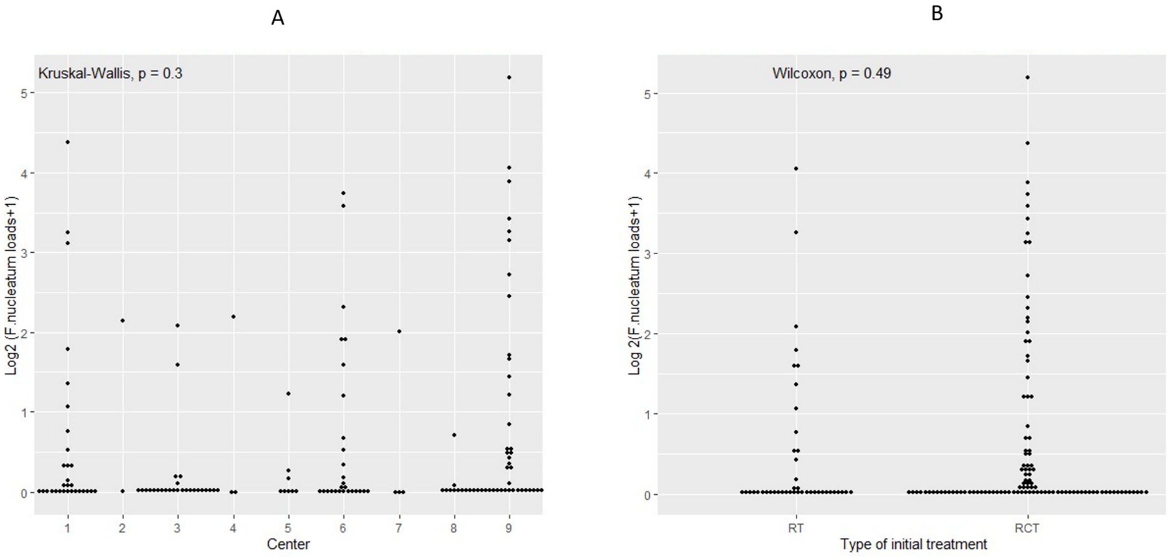

Figure S2. Distribution of normalized Fusobacterium loads. Distribution of logged Fusobacterium loads according to individual centers (**A**) and the type of initial treatment (**B**).

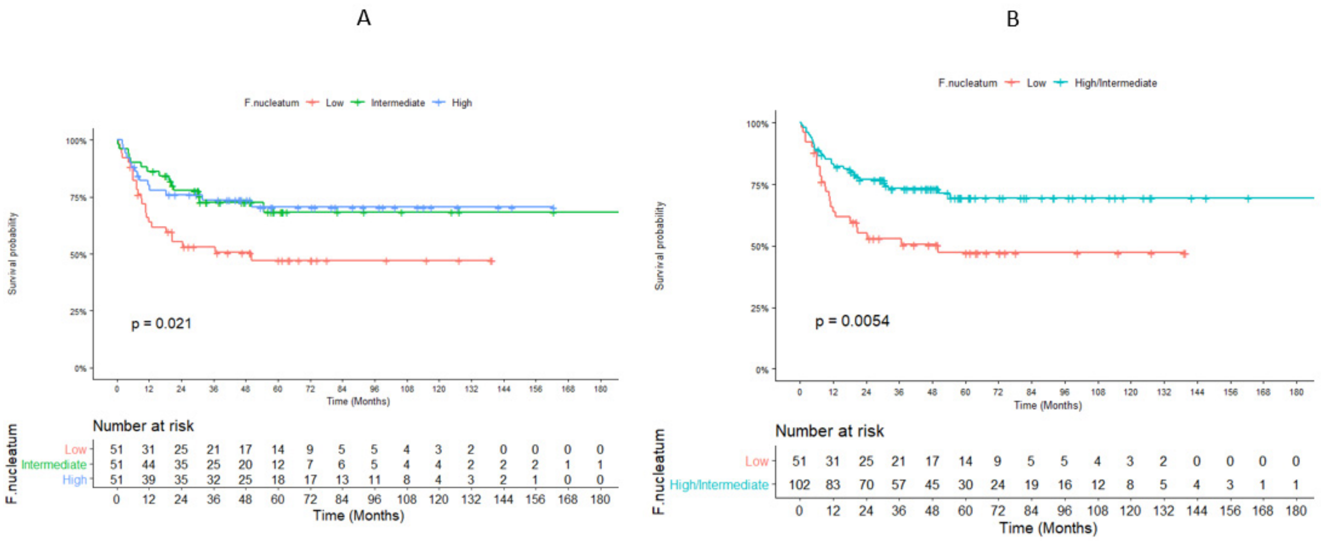

Figure S3. Association between metastasis-free survival and *Fusobacterium nucleatum*. Metastasis-free survival curves for the *Fusobacterium nucleatum* divided in 3 categories according to terciles (**A**) and 2 categories according to terciles (**B**), n = 153 patients.

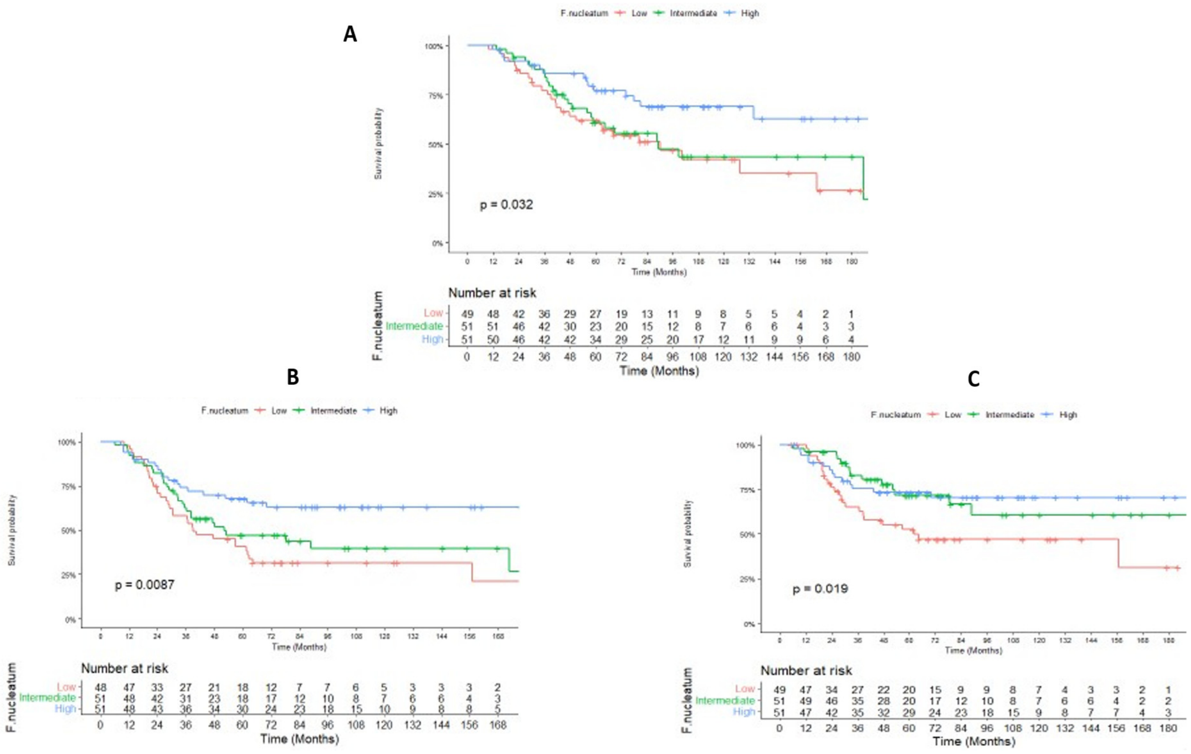

Figure S4. Association between survival with the diagnosis taken as starting point and *Fusobacterium nucleatum*. Overall-free survival (**A**), disease-free survival (**B**) and metastasis-free survival (**C**) curves for the *Fusobacterium nucleatum* divided in 3 categories according to terciles.

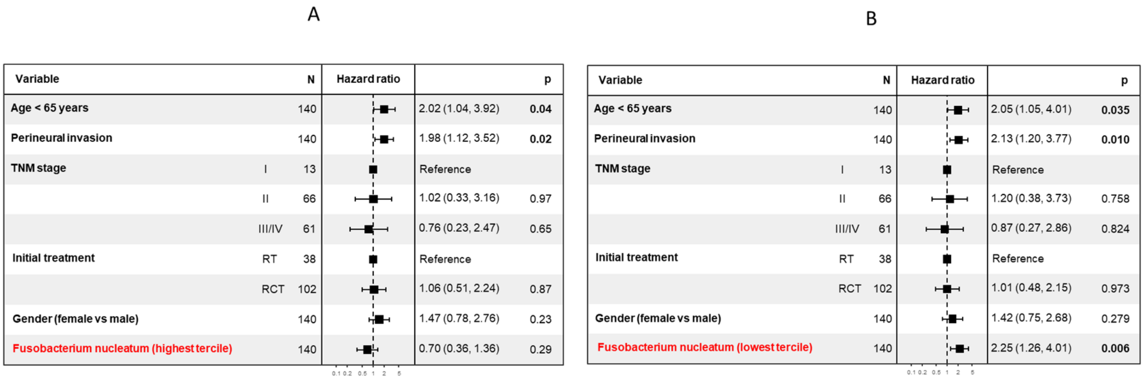

Figure S5. Prognostic value of clinicopathological factors and Fusobacterium nucleatum. Multivariate analysis for the clinicopathological factors regarding metastasis-free-survival (*n* = 150 patients), with the highest tercile (**A**) or lowest tercile (**B**) as a reference for Fusobacterium loads.
